# Texture Analysis Using Indocyanine Green Fluorescence Measurement During Porcine Hypothermic Oxygenated Machine Perfusion

**DOI:** 10.1111/aor.70199

**Published:** 2026-08-28

**Authors:** Yuga Okazawa, Hiromichi Obara, Taiki Mori, Satoshi Muto, Mio Takashima, Masafumi Furukawa, Hiroki Kaneko, Tetsuya Nakajo, Shohei Ishizawa, Seisuke Sakamoto, Naoto Matsuno

**Affiliations:** ^1^ Tokyo Metropolitan University Tokyo Japan; ^2^ Azabu University Veterinary Teaching Hospital Sagamihara Japan; ^3^ One for Animals Tokyo Saitama Japan; ^4^ Nippon Veterinary and Life Science University Tokyo Japan; ^5^ National Center for Child Health and Development Tokyo Japan

**Keywords:** HMP, ICG, liver, machine perfusion, viability assessment, gray‐scale level co‐occurrence matrix (GLCM)

## Abstract

**Background:**

Development of novel organ viability assessment methods during MP under hypothermic conditions is important to expand the donor pool with DCD donors for transplantation. This study proposed a novel quantitative method using Indocyanine Green (ICG) fluorescence image for assessing organ viability during hypothermic oxygenated machine perfusion (HOPE), specifically focusing on homogeneous flow distribution analyzed with the gray‐scale level co‐occurrence matrix (GLCM) method, which is one of the most effective texture analysis method.

**Method:**

The proposed method quantifies ICG image uniformity using GLCM's ‘contrast’ feature, defining an evaluation index *h* as the average contrast (𝑐𝑜𝑛𝑡𝑟𝑎𝑠𝑡_𝑎𝑣𝑒_) after 200 s of ICG inflow. Experiments involved porcine livers with 0 min (WIT0) or 45 min (WIT45) of warm ischemia, preserved by dual‐hypothermic oxygenated machine perfusion (HOPE), then evaluated using autologous blood reperfusion under normothermic conditions with an Isolated Reperfusion Model (IRM).

**Results:**

Qualitatively, WIT45 livers showed more heterogeneous perfusion with “localized non‐fluorescent regions”. Quantitatively, the evaluation index *h* indicated a strong positive correlation with portal vein resistance at 120 min during IRM (*r* = 0.70, *p* = 0.015). A correlation with hepatic artery (HA) resistance (*r* = 0.40) was also investigated. This method enables quantitative prediction of ischemic injury status before transplantation, which is crucial for making informed decisions about DCD and marginal organs. It could expand the possibilities for HOPE perfusion.

**Conclusion:**

In conclusion, the evaluation index h obtained from GLCM analysis of ICG fluorescence images in organs during MP can predict portal vein resistance induced by ischemic reperfusion injury before transplantation and is a method that can expand the possibilities of HOPE perfusion.

## Graphical abstract

A novel quantitative method using Indocyanine Green (ICG) fluorescence image for assessing organ viability during hypothermic oxygenated machine perfusion (HOPE), specifically focusing on homogeneous flow distribution analyzed with the gray‐scale level co‐occurrence matrix (GLCM) method, can predict portal vein resistance induced by ischemic reperfusion injury before transplantation.

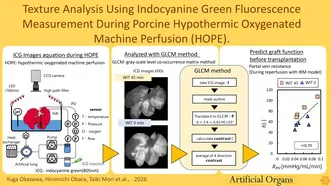

## 1 Introduction

Expanding the donor pool by utilizing marginal grafts donated after circulatory death (DCD) is a potential solution to the global shortage of donors. The use of DCD donors for transplantation requires preventing the high risk of primary nonfunction (PNF), biliary complications, and ischemic reperfusion injury due to warm ischemia [1, 2]. Machine perfusion (MP) is a key technology for both restoring and evaluating the function of donor organs, thereby extending donor criteria. There are three strategies: normothermic regional perfusion (NRP) [3, 4, 5], normothermic machine perfusion (NMP) [6, 7] and hypothermic machine perfusion (HMP) [8]. MP has been reported to reduce the incidence of non‐anastomotic biliary strictures (NAS) and primary nonfunction (PNF) more than the conventional static cold storage (SCS) method [6, 7, 8, 9, 10]. Although NMP is a promising technology for DCD donors [7], the complex perfusion system has considerations regarding the maintenance costs and management risks. On the other hand, HMP is perfused at low temperatures (7°C–10°C) to preserve organ functions and maintain low metabolism. Hypothermic oxygenated machine perfusion (HOPE) is a perfusion method that operates at low temperatures with oxygenation, aiming to reduce the incidence and severity of ischemia–reperfusion injury and improve outcomes after organ transplantation [11]. While HMP and HOPE have promising benefits in terms of preserving organs, the evaluation methods for organ viability under hypothermic conditions are not yet sufficiently established. It is important to develop novel organ assessment methods during MP under hypothermic conditions.

Flavin mononucleotide (FMN) leakage into the perfusate during MP can be used as an organ function assessment method to evaluate mitochondrial dysfunction before transplantation [12, 13]. Currently, FMN is considered a powerful evaluation method; however, the existence of multiple evaluation methods from different perspectives enables the establishment of reliable transplantation. In particular, the quantitative evaluation of fluid flow within organs is considered extremely useful in predicting ischemia–reperfusion injury.

Indocyanine green (ICG) fluorescence images have significant potential not only to evaluate liver homogeneous perfusion conditions qualitatively but also to identify liver function quantitatively. ICG binds to albumin and lipoproteins and exhibits fluorescence properties. In this study, ICG is used as a tracer dye based on its near‐infrared fluorescence properties. As long as blood flow is maintained, ICG can be delivered to the hepatic lobules via the blood vessels, regardless of the degree of metabolism. Conversely, areas where the dye does not reach are regions with impaired perfusion; thus, the proposed method allows quantification of perfusion heterogeneity. It is widely used in clinical settings for imaging the vascular system during surgery [14, 15]. Gyung Mo Son et al. evaluated the hemodynamics of anastomotic regions by focusing on the time variation of ICG fluorescence [16], and Figueroa et al. concentrated on the pattern of ICG fluorescence in the liver after transplantation [17]. However, these evaluations are qualitative and dependent on environmental factors such as illumination. Furthermore, Goto et al. were employed to assess organ quality with ICG measurement for organ preservation [18] The use of thresholds in methods often presents numerous challenges when employed as conventional approaches for adjusting lighting intensity and shooting conditions. A novel analytical method is essential for developing an evaluation method of organ function using ICG measurement during MP.

We considered utilizing texture analysis to evaluate fluid flow within organs during preservation using MP prior to transplantation. The term “texture” originally referred to the pattern of folds in fabric and describes the surface quality of a material. The texture of an object's surface is expected to provide a wealth of information for object identification, such as roughness, periodicity, and randomness [19]. While various methods of texture analysis have been proposed, the Gray Level Co‐occurrence Matrix (GLCM) is a technique capable of quantifying mottled patterns and is increasingly being utilized across various fields. For example, it is an indicator adopted as a feature in The Image Biomarker Standardization Initiative [20] as part of efforts to standardize the use of radiological images for diagnosis, and it is considered applicable to the evaluation of organs using near‐infrared images. Proposed by Haralick [21] et al., GLCM is a method that compares the luminance values of any two pixels in an image; by calculating features, it quantitatively evaluates image uniformity and other characteristics. Furthermore, because it compares only image information within the same image, GLCM has the potential to be evaluated independently of lighting conditions or the imaging environment.

In this study, we proposed and evaluated a quantitative organ viability assessment method using the gray‐scale level co‐occurrence matrix (GLCM) method, which is one of the most effective texture analysis methods, to assess a homogeneous flow state of the perfused organ with ICG fluorescence measurement during HOPE before transplantation.

## 2 Methods

### 2.1 Animals

Nine Female, cross‐bred, Large White, Landrace, and Duroc pigs, 2–3 months of age and approximately 20–30 kg of body weight, were supplied by Tokyo Laboratory Animals Science Co. Ltd. All animal work was conducted according to the relevant conditions. The research protocol was approved by the Institutional Animal Ethics Committee of the Clinical Research Center, National Center for Child Health and Development (permit No. 2021–001‐CO1).

### 2.2 Preparation and Preservation

The pigs were intubated and ventilated under inhalation anesthesia with isoflurane (Forane VR; Abbot Japan, Tokyo, Japan). A midline abdominal incision was made. The pigs were injected with potassium chloride intravenously to induce cardiac arrest, followed by ventilation removal, as described previously in detail [22]. The induction of cardiac arrest was determined as the starting point of warm ischemia.

### 2.3 Machine Perfusion Device

In this study, dual‐HOPE, which perfused both the portal vein (PV) and the hepatic artery (HA), was performed using the original device developed in our laboratory (Figure 1). The details of the perfusion system are described in a previous report [23, 24]. A brief description is given here. The two perfusion circuits for portal vein (PV) and hepatic artery (HA) have pressure sensors (KL76; Nagano Keiki, Nagano, Japan), flow meters (PV: VN05; Aichi Tokei, Japan, HA: FD‐SS02; Keyence, Japan), thermocouples, and oxygen sensors (NeoFox; Ocean Optics, USA). Oxygen was supplied to the perfusate using a membrane oxygenator (HP0‐06 H‐C; Senko Medical Instrument, Tokyo, Japan), and the oxygen sensors were installed in the PV circuit and perfusate correction port. The perfusion conditions were set to target HA, and PV flow rates were controlled at 0.22 and 0.06 mL/min/g, respectively [24]. Modified Histidine‐Tryptophan‐Ketoglutarate (HTK) solution was used as the perfusate, which previous research has demonstrated the potential for its use as a liver perfusion solution [25], and the perfusate included albumin to enhance the fluorescence of ICG. The perfusate temperatures were maintained at 7°C–10°C during the hypothermic condition. The oxygenator was regulated at FiO_2_: 0.4, Gas Flow: 2 L/min.

**FIGURE 1:**
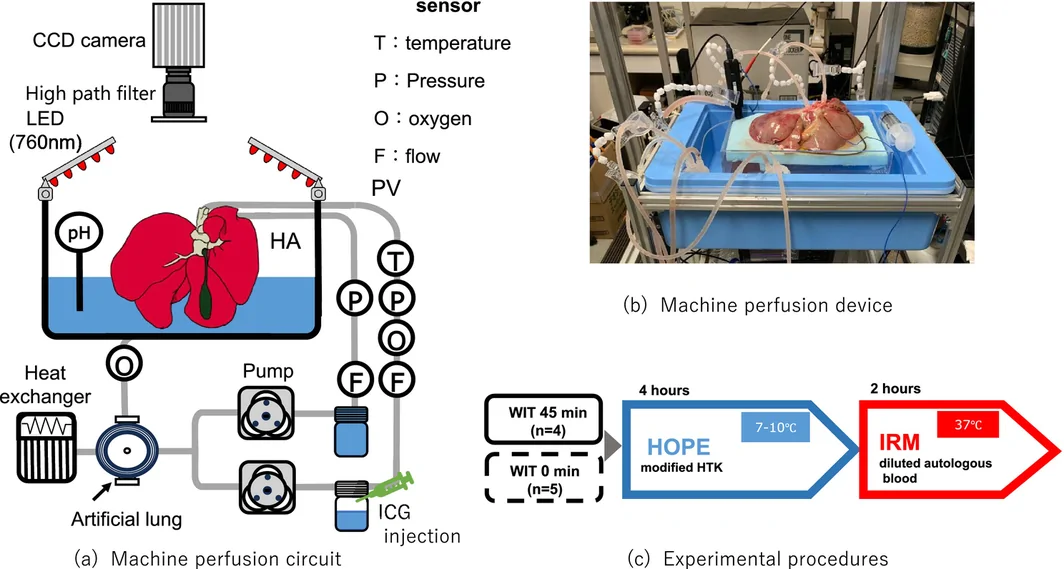
Device of Machine Perfusion (a, b) and Experimental design (c) WIT: Warm Ischemic Time, HOPE: Hypothermic Oxygenated Machine Perfusion, IRM: Isolated Reperfusion Model.

### 2.4 Isolated Reperfusion Model (IRM)

The isolated reperfusion model (IRM) [26] was employed to evaluate the preserved liver graft function after HOPE. IRM imitates a reperfusion situation after transplantation using 1.2 L diluted autologous blood under normothermic temperature conditions (35°C–37°C). This method was designed to confirm episodes occurring in the short term, such as ischemia–reperfusion injury. This method allows evaluation of organ functions following preservation during a relatively short reperfusion period of approximately 2 h. While assessing organ metabolic characteristics relevant to long‐term prognosis requires a longer evaluation period using whole blood, this method can be performed with diluted blood; it is therefore a useful technique due to its ease of management and is also beneficial from an animal welfare perspective. Autologous blood was collected in small increments until a total of 400 mL was obtained, while maintaining porcine hemodynamic stability. Detailed procedures of reperfusion were described in previous papers [26, 27]. Heparin was added to the reperfusion solution, and the activated clotting time (ACT) was adjusted to less than 200 s. Hematocrit values were maintained between 10% and 12%. The oxygenator was regulated to achieve physiological blood gas values (pO_2_, approximately 150–200 mmHg; pCO_2_, approximately 30–50 mmHg) [25].

### 2.5 Experimental Design

Figure 1c shows the experimental design. Porcine liver grafts (weight 763 ± 136 g before perfusion) had a warm ischemia time (WIT) of 45 min (*n* = 4) or no WIT (*n* = 5). After removal, 1 L of EuroCollins, which contains heparin, was used for washout through PV and HA. Both groups consisted of Dual‐HOPE at 7°C–10°C for 4 h. During dual‐HOPE, ICG was administered exclusively via the portal vein to ensure rapid delivery to the hepatic lobules. We used ICG (DIAGNOGREEN FOR INJECTION (25 mg), DAIICHI SANKYO, Japan), which was aliquoted into 2.5 mg doses and diluted with 1 mL of saline (2.5 mg/mL). At 180 min after the start of HOPE, 0.2 mL was injected into a container holding the fluid from the portal circulation, ensuring it was gently perfused. ICG fluorescence images were captured by a high‐sensitivity CCD camera (Image EM X2; Hamamatsu Photonics, Japan) illuminated with near‐infrared LED light unit (760 nm, in‐house device). Finally, both groups were evaluated with IRM for 2 h using diluted autologous blood under normothermic temperature conditions (35°C–37°C).

### 2.6 Evaluation During IRM

Aspartate aminotransferase (AST) and lactate dehydrogenase (LDH) levels in the perfusate were measured during IRM to assess viability. Serum glucose and lactate concentrations were measured using a handheld blood analyzer (iSTAT, Abbott, USA). PV and HA resistances were calculated by dividing the pressure value by the flow rate. Lactate clearance was calculated based on its change during blood reperfusion.

### 2.7 ICG Fluorescence Image During HOPE Analysis

In this study, we focused on an analysis method called the gray‐scale level co‐occurrence matrix (GLCM), which was proposed by Haralick et al. [21]. GLCM is a method for quantitatively evaluating the uniformity and other characteristics of an image by calculating feature values. An example of an actual calculation process is shown in Figure 2a. Figure 2b is a matrix of the luminance values of a sample image. Figure 2c,d are matrices of the luminance values of the pixel of interest and the pixel to be compared. Then, the distance *d* and angle θ of the compared pixels are set, and each pixel is compared. The angles that can be set are 0°, 45°, 90°, and 135° (Figure 2). Comparisons are performed on both sides of the set angles θ and θ + 180°. For example, if *d* = 1 and θ = 0° in Figure 3b, there are two combinations where the pixel of interest and the pixel to be compared have a luminance value of 0, and 2 is entered in (0,0) in Figure 2b. This is done for each pixel, and the matrix is shown in Figure 2b. Figure 2d is the sum of Figure 2c divided by the sum of Figure 2c, and shows the probability that the combination will occur. This is called the GLCM.

**FIGURE 2:**
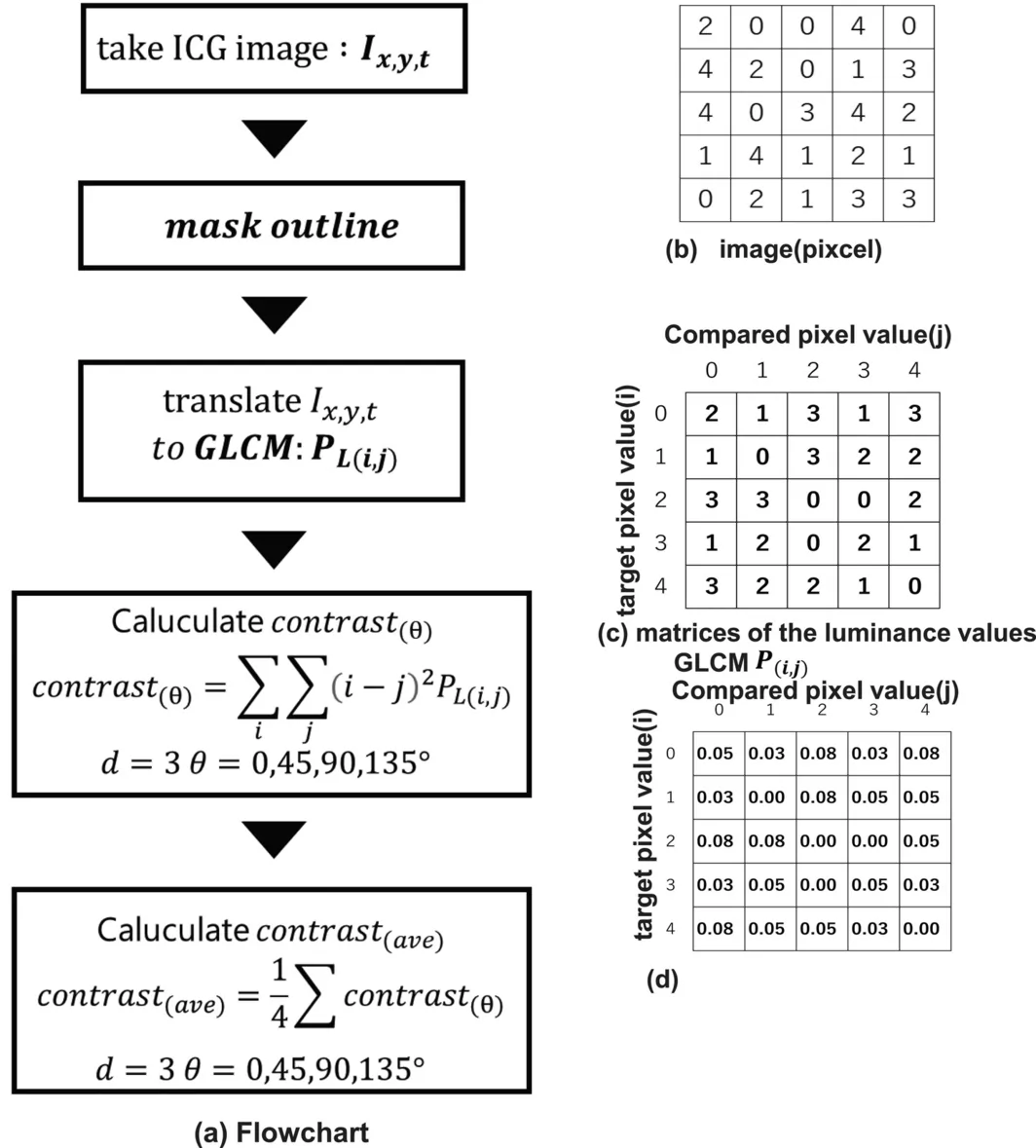
Procedure of GLCM analysis and Process of conversion from image to GLCM (*d* = 1, *θ* = 0°).

**FIGURE 3:**
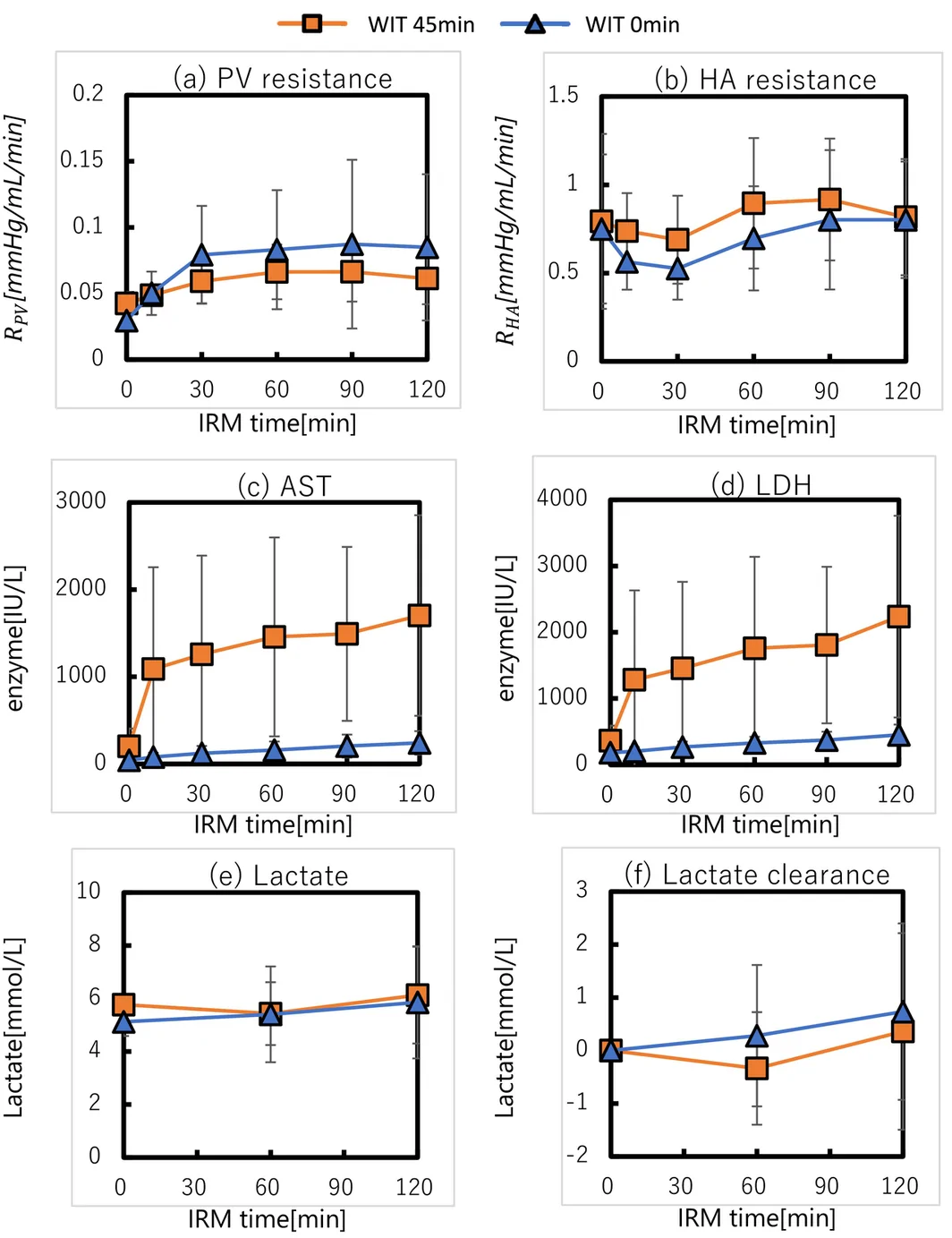
Result of IRM (isolated reperfusion model). Portal Vein Resistance (b) Hepatic artery resistance (c) AST (Aspartate aminotransferase) (d) LDH (lactate dehydrogenase) (e) Lactate clearance (time variation from IRM start).

The uniformity features of the GLCM are explained [21, 28, 29]. $P_{\left(i,j\right)}$ and i are the luminance values of the pixel of interest, and *j* is the luminance value of the pixel being compared. $P_{\left(i,j\right)}$ were calculated following equations (1) and (2).

(1) $$C_{d,\theta }\left(i,j\right)=\sum_{x=1}^{\mathrm{Nx}}\sum_{y=1}^{\mathrm{Ny}}\left\{\begin{array}{c} 1\;\text{if}\;I\left(x,y\right)=i\;\text{and}\;I\left(x+\mathrm{\Delta x},y+\mathrm{\Delta y}\right)=j\\ 0\;\text{otherwise} \end{array}\right.$$

(2) $$P\left(i,j\right)=C_{d,\theta }\left(i,j\right)/\;\sum_{i=0}^{G-1}\sum_{j=0}^{G-1}C_{d,\theta }\left(i,j\right)$$

Equation (3) is called contrast and is the sum of the GLCM multiplied by the square of the difference in luminance values; if the luminance values are similar, contrast will be low. In this study, the contrast in equation (1) was used as a feature to evaluate uniformity.

The procedure for GLCM analysis in this study is illustrated in Figure 2. Masking was performed on the captured images using the liver contour, and the fluorescence information of the liver only was retained (the brightness values of the non‐liver images were set to 0). Python was used to calculate the GLCM $P_{\left(i,j\right)}\left(0\leq i\leq 255,0\leq j\leq 255\right)$. Here, since $P_{\left(i,j\right)}$ contains information other than the liver. The first row and the first column of $P_{\left(i,j\right)}$ were deleted. Then, normalized ${\text{contrast}}_{\left(\theta \right)}$ was calculated from $P_{\left(i,j\right)}$. In this study, *d* = 3 was set based on the calculation cost and the resolution of the ICG fluorescent images to represent the size of a hepatic lobule. To eliminate the dependence of the arranged livers, the calculation was performed in four directions (*θ* = 0, 45, 90, 135°). Finally, the average of the calculated ${\text{contrast}}_{\left(\theta \right)}$ in four directions was defined as ${\text{contrast}}_{\mathrm{ave}}$, and the possibility of quantitative evaluation was examined.

(3) $$\text{contrast}=\sum_{i}\sum_{j}{\left(i-j\right)}^{2}P_{\mathrm{ij}}$$

The *contrast*_ave_, which allows for the quantitative representation of mottled and homogeneous states within an organ, can be obtained from each acquired image; by evaluating its temporal changes, it becomes possible to quantitatively discuss changes in the characteristics of microcirculation in the hepatic lobule. In this report, we defined the average value after the time at which this change reaches a steady state as the evaluation index h, and used it as an indicator to evaluate microcirculation in each experiment. Note that, as described later in this report, the evaluation index h was calculated as the average of the values that had stabilized 200 s or more after ICG administration.

### 2.8 Statistical Analysis

The results are presented as mean ± SD. The data were analyzed using R version 4.4.2. Statistical significance difference was *p* < 0.05.

## 3 Results

### 3.1 HOPE Perfusion Parameters

Flow rate, pressure and resistance values during HOPE for PV were 111 ± 7 versus 108 ± 7 mL/min (n.s), 4 ± 1 versus 5 ± 1 mmHg (n.s), 0.03 ± 0.01 versus 0.04 ± 0.01 mmHg/mL/min (n.s). And for HA were 44 ± 5 versus 57 ± 4 mL/min (n.s) and 16 ± 7 versus 21 ± 2 mmHg (n.s), 0.37 ± 0.17 versus 0.37 ± 0.06 mmHg/mL/min (n.s). (WIT45 vs. WIT0, respectively).

### 3.2 Evaluation of Organs by Blood Reperfusion

Figure 3 shows the graphs of PV resistance, HA resistance, AST, LDH, lactate, and lactate clearance during blood reperfusion with the IRM model. The results are for 120 min after the start of blood reperfusion. Figure 3a PV resistance showed little difference between groups immediately after the start of IRM (0.04 ± 0.01 vs. 0.03 ± 0.01, *p* = 0.09), although PV resistance was slightly higher toward the end of IRM. (a) HA resistance was not significantly different between groups (0.06 ± 0.02 vs. 0.08 ± 0.06, *p* = 0.46), (b) HA resistance tended to be higher in WIT45 immediately after the start of IRM (0.79 ± 0.50 vs. 0.75 ± 0.42, *p* = 0.91), and no significant difference was shown at the end of IRM (0.82 ± 0.33 vs. 0.80 ± 0.33, *p* = 0.96). AST and LDH, deviating enzymes during blood reperfusion, tended to be higher in WIT 45 but ASTdid not show significant differences even at the end of IRM due to large variability (1702 ± 1150 vs. 241 ± 135, *p* = 0.11). LDH also showed a similar difference in the mean value, but also with considerable variability. LDH did not indicate a significant difference (2237 ± 1522 vs. 452 ± 153, *p* = 0.13). Lactate and lactate clearance showed a slight tendency to be lower in WIT0, but there was no significant difference between groups (6.13 ± 1.83 vs. 5.86 ± 2.12, *p* = 0.86). The lactate clearance from the beginning to the end of IRM showed an increasing trend for WIT45 and a decreasing trend for some WIT0 (0.36 ± 1.85 vs. 0.73 ± 1.67, *p* = 0.86), although no significant differences were observed.

### 3.3 ICG Fluorescence Images

Here, the ICG fluorescence images measured during dual‐HOPE, used for data analysis, are presented in a time series in Figure 4. Representative ICG fluorescence images of each group are shown here. The time intervals between sequential images are not equal. The results are presented up to 600 s from the start of ICG inflow into the organ. Fluorescent regions indicate areas of inflow of ICG excited by NIR LEDs, and non‐fluorescent regions indicate areas of no ICG inflow. The fluorescence images show that the area of fluorescence expanded over time. Both groups showed non‐fluorescent regions at the end of the liver. The non‐fluorescent area was primarily located on the left lobe of the liver. Comparing both groups, localized non‐fluorescent regions were observed in the WIT45 group, and more heterogeneous perfusion areas were observed in the WIT45 group than in the WIT0 group. On the other hand, the WIT0 group showed no localized non‐fluorescent areas and more uniform fluorescent areas. Qualitatively, organs in poor condition (WIT 45 min) with long warm ischemic time showed more non‐uniform speckled areas, while organs in good condition (WIT 0 min) showed uniform fluorescent regions.

**FIGURE 4:**
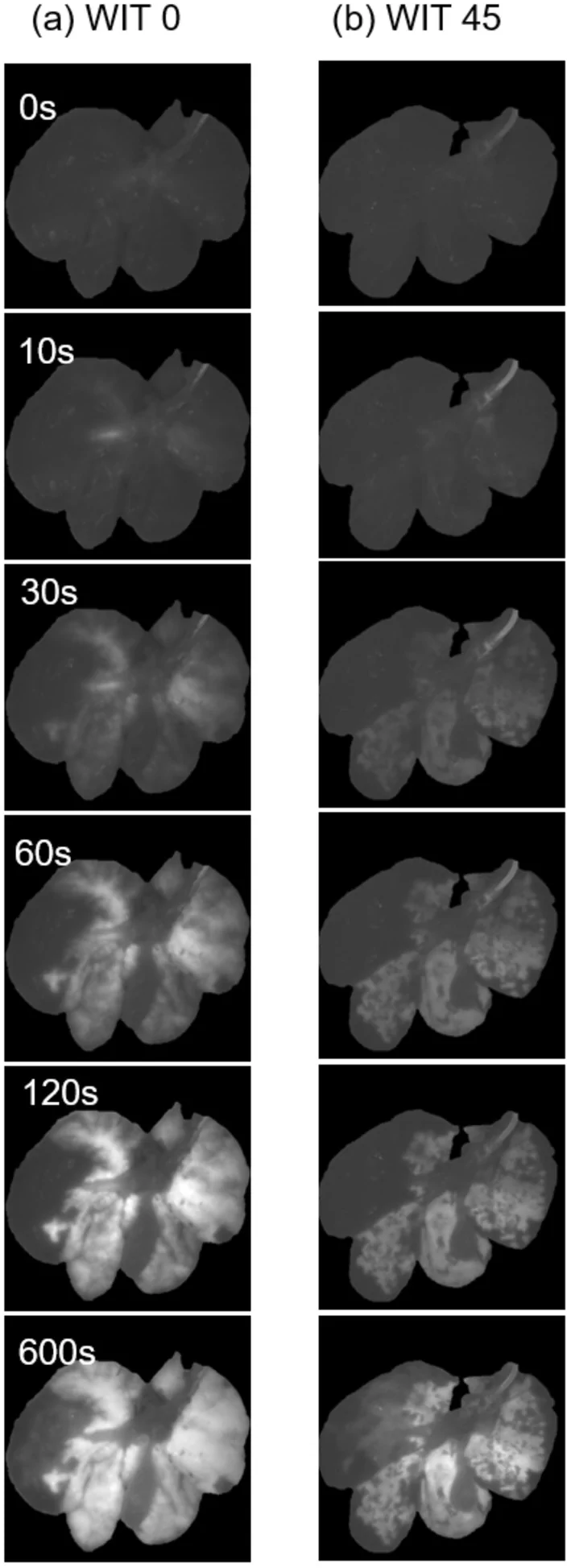
Image of ICG fluorescence during HOPE.

### 3.4 GLCM Contrast Average

Figure 5 shows the results of the GLCM analysis in four directions using the ICG fluorescence images. The average of the four directions, $\text{contras}t_{e}$, is calculated. (a) The horizontal axis shows the time from the initial timing of the ICG inflow, corresponding to the images described in Figure 4. The vertical axis is the $\text{ontras}t_{\mathrm{ave}}$ calculated with the GLCM method shown in Figure 2 and, corresponding to the speckled state of the image at each time point. In both groups, $\text{ontras}t_{\mathrm{ave}}$ increases with time and remains constant at about 200 s. As shown in the image mentioned above (Figure 4), what was shown as heterogeneous perfusion in the WIT45 group is shown as quantitative information by $\text{contras}t_{e}$. And these values tend to be higher than WIT0 at all times. From the results of Figures 4 and 5, we assume that the situation where fluorescence shows a steady state after 200 s. The average value of $\text{contras}t_{\mathrm{ave}}$ after 200 s is defined as the evaluation index *h* and will be used for the evaluation in the subsequent detailed analysis.

**FIGURE 5:**
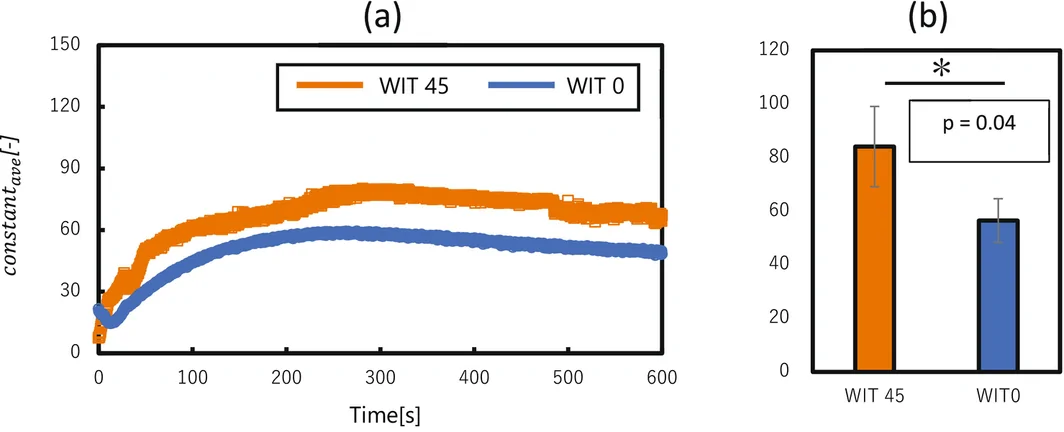
Result of ICG fluorescence image analysis with GLCM (a) *contrast*_*ave*_ (b) h (average of *contrast*_*ave*_) after 200 s ICG injection.

In Figure 5b, we present a comparison of the evaluation index *h* between groups. Comparing WIT0 and WIT45, the WIT0 group had lower *h* values, and WIT45 often showed confinement, similar to the qualitative image information. The evaluation index *h* shows significant differences (84.11 ± 14.97 vs. 56.50 ± 8.14, *p* = 0.04).

### 3.5 Correlation Between the Results of Blood Reperfusion and the Evaluation Index *h*

Figure 6 illustrates the correlation between the evaluation indices derived from the analysis of ICG fluorescence images acquired during MP and the conventional endpoints measured during blood reperfusion using the IRM method.

**FIGURE 6:**
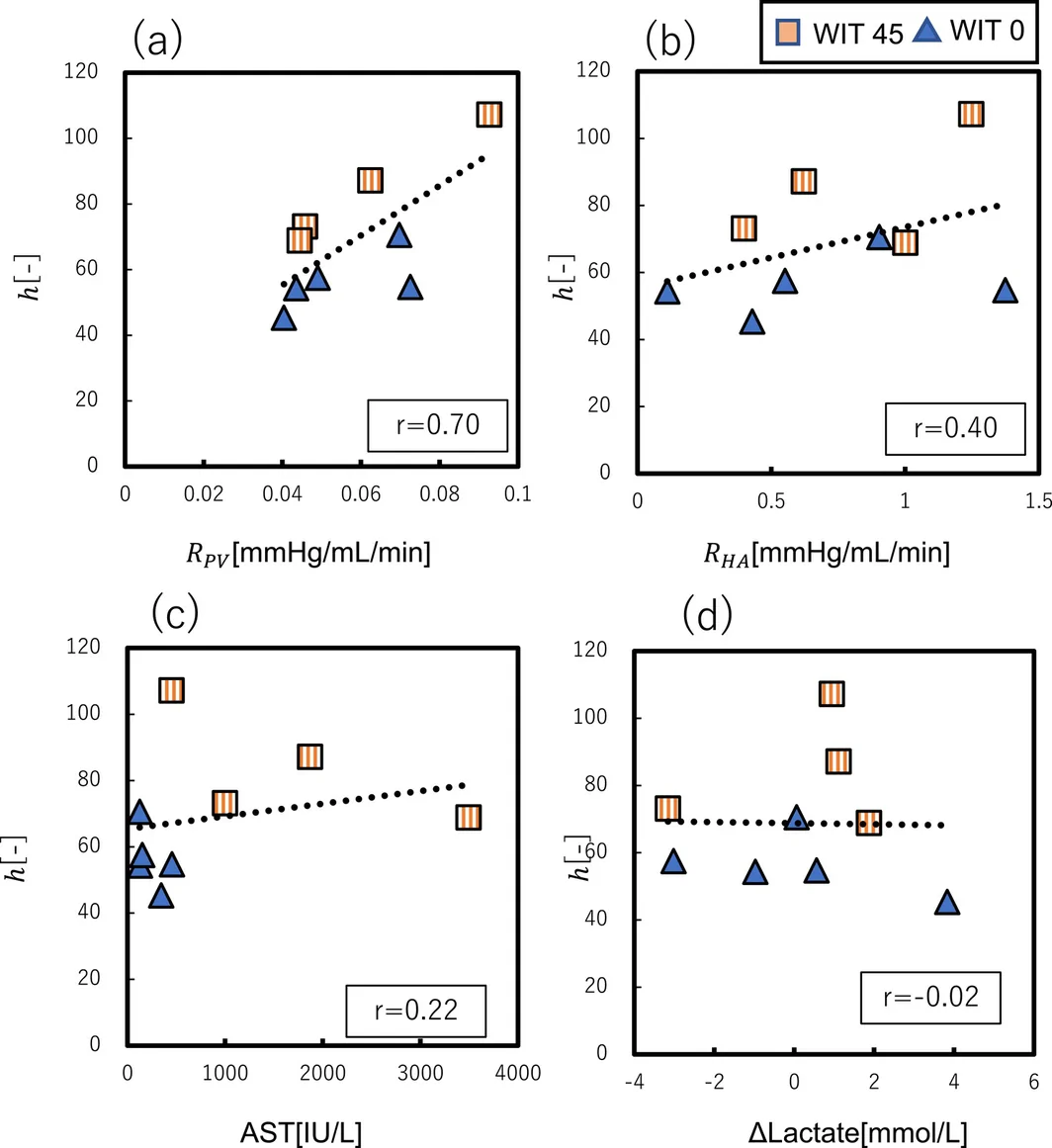
Correlation of h and AST, lactate clearance during IRM. (a): IRM 120 min PV resistance (b): IRM 120 min HA resistance (c): IRM 120 min AST (d): IRM 120 min time variation from IRM start.

A strong positive correlation was observed between the evaluation index h and PV resistance (*r* = 0.85, *p* = 0.015). The correlation is even stronger in the WIT45 and WIT0 groups. In HA resistance, a correlation is shown (*r* = 0.69). No correlations were shown with the abscissa AST (*r* = 0.11), lactate clearance (*r* = 0.02).

## 4 Discussion

A quantitative organ viability assessment method was introduced to evaluate the homogeneous flow state of the perfused organ using ICG fluorescence image measurement and the GLCM (gray‐scale level co‐occurrence matrix) method during HOPE before transplantation. ICG is used as a tracer dye based on its near‐infrared fluorescence properties, and the GLCM method allows quantification of perfusion heterogeneity. We demonstrated that analyzing ICG fluorescence images using the GLCM method—one of the texture analysis techniques proposed in this report—enables the prediction of portal vein pressure during reperfusion, a condition that can be considered one of the outcomes of ischemia–reperfusion injury prior to reperfusion.

MP is a promising method for addressing the donor shortage problem in transplantation and is indispensable, especially for DCD donor transplantation. The risk of primary nonfunction (PNF), biliary complications, and ischemic cholangiopathy has to prevent the use of DCD donors for transplantation [30, 31] due to warm ischemia. Although the clinical use of NMP with established procedures for organ viability assessment has led to a dramatic increase in the number of transplanted livers from DCD donors in the USA, the higher cost and complex management of perfusion with blood at normothermic temperatures are considerations for its routine clinical use [32, 33]. On the other hand, HMP and HOPE have even higher expectations for preservation of organs due to their simplicity and ease of use [8, 9, 10]. However, there are still many debates regarding the viability assessment of livers during HMP or HOPE, despite great expectations for the possibility of evaluation by FMN [34, 35, 36, 37]. In the case of measurements using perfusion fluid, as with FMN, the leakage amount may be significantly affected by the perfusion state of the organ, and it is considered necessary to evaluate not only the simple concentration but also the perfusion state. It is essential to develop evaluation techniques that can be used not only with FMN measurement technology but also with supporting technology in low‐temperature perfusion conditions.

Quantitatively evaluating fluid dynamics within organs is crucial for assessing organ function. Fluid dynamics disorders within organs are one form of ischemia–reperfusion injury. In previous NMP studies, several indicators have been proposed for evaluating organ function, and their usefulness is being validated through follow‐up in clinical studies. While biomarkers such as lactate levels are important, other indicators such as vascular resistance and perfusion uniformity are also considered crucial. However, some aspects of organ fluid dynamics, such as uniformity, are difficult to evaluate quantitatively, making quantitative indicators for assessing fluid uniformity indispensable. If it is possible to quantify the uniformity of fluid flow distribution within organs, this could contribute significantly to the evaluation of organ viability before transplantation. The relationship between ischemia–reperfusion injury and intra‐organ blood flow has long been a subject of debate [38, 39], and it has already been discussed that dysfunction can occur due to hepatic microcirculatory perfusion failure [39]. However, conventional evaluation methods often rely solely on flow rate, pressure, and the resistance calculated from these parameters. Although studies focusing on dynamic changes in flow, such as temporal variations, have been reported [40, 41]. There has been insufficient quantitative discussion regarding the distribution of these changes. This method proposes a technique for image measurement—which has traditionally relied on qualitative evaluation—that quantifies spatial information regarding flow characteristics by focusing on texture analysis rather than threshold‐based evaluation, thereby enabling the clarification of flow characteristics during organ reperfusion within the MP.

ICG fluorescence image measurement has the potential to evaluate the uniformity of fluid flow distribution within organs. ICG fluorescence images have significant potential not only to evaluate liver homogeneous perfusion conditions qualitatively but also to identify liver function quantitatively. ICG is well known as a conventional measurement tool to investigate liver function. Also, ICG fluorescence images are employed as a valuable marker to identify the organ region for surgical treatment [16, 17]. There are some studies that not only qualitatively evaluate liver homogeneous perfusion conditions but also quantitatively identify liver functions. ICG was employed to assess organ quality with ICG image measurement for organ preservation [18]. The use of thresholds in evaluation methods often presents numerous challenges when employed as conventional approaches for adjusting lighting intensity and illuminating conditions. A novel analytical method is essential for developing an evaluation method of organ function using ICG measurement during MP. Methods focusing on temporal changes in brightness [42] have demonstrated that brightness changes at 150 s can provide good predictions of transplant outcomes from 76 cases of intra‐organ flow, and this paper strongly supports the potential use of ICG. On the other hand, spatial image information also contains significant value, and this report presents a key approach for utilizing spatial ICG fluorescence information. We believe that methods combining the evaluation of both spatial and temporal information will become increasingly important in the future.

The proposed method is intended to enable the determination of organ function during MP before transplantation. Before validating the proposed method, the results were obtained during blood reperfusion of organs preserved by MP, which served as the basic data for method validation. The results were obtained using IRM, which can evaluate the state of preservation of organs with reperfusion. The vascular resistance and lactate clearance have been adopted as evaluation indices of organ function in the NMP, which is now widely used clinically as criteria of transplantation [7]. The fact that no differences were observed between the WIT0 and WIT45 groups in Figure 3. Some organs of WIT45 may have undergone functional recovery through HOPE. In this study, we chose to assess the effectiveness of this measurement method using correlation values instead of group data comparison.

Figure 6 shows a strong correlation between the vascular resistance during blood reperfusion and the evaluation index *h* acquired during MP. This correlation may indicate that the ischemic injury situation in organs can be quantitatively predicted before transplantation. Here, we compared the correlation between the evaluation index *h* and various factors measured during blood reperfusion. These indices are well‐established quantitative functional assessment indicators of NMP [7]. In particular, lactate clearance has become an important indicator and a demand item for clinical evaluation. In this study, a strong correlation was demonstrated between the evaluation index *h* and vascular resistance, but a weak relation was found with lactate clearance. The detailed relationship between metabolic factors, such as lactate clearance, and fluid factors, such as HA resistance and index h, needs to be discussed further in the future.

The evaluation index *h* is a method that can quantitatively evaluate the uniformity of image information obtained by ICG fluorescence image measurement using the GLCM method. The GLCM method is a promising technique for not only texture analysis but also medical evaluation tools [28, 29]. In other words, it is a method that can quantify non‐uniformity, such as speckles. In Figure 6, WIT0 grafts in good condition and poor condition WIT45 are compared. As images, we showed differences in the trend of ICG fluorescence distribution. This suggests that there is also a difference in the flow distributions within the organ, indicating a flow disturbance within the organ compared to an organ with good WIT0. Organs with longer WIT are assumed to suffer ischemic injury, and the qualitative status of that damage can be observed after blood reperfusion, before ischemia–reperfusion damage is shown.

Ischemia reperfusion injury is associated with a vascular phenotype that includes increased vascular permeability, endothelial cell inflammation, an imbalance between vasodilating and vasoconstricting factors and activation of coagulation and the complement system [43]. Marked sinusoidal congestion occurs during ischemia–reperfusion injury, accompanied by microvascular spasm, swelling of endothelial cells, bleb formation on the endothelial surface, increased capillary permeability, interstitial edema, micro‐thrombi, neutrophil adhesion and plugging, local acidosis, and presence of red blood cells with altered micro‐rheology [39, 44]. In the WIT‐treated group, hemorrhage and blood flow arrest were observed in the hepatic lobes and sinusoids. In addition, Iwata et al. reported histological evidence of congestion and cytoplasmic emptying in the sinusoids after 45 min of WIT, porcine livers were preserved in HOPE [22], and we believe that similar results were obtained in the present study. Furthermore, the time variation of the value of $\text{contras}t_{\mathrm{ave}}$ indicates that ICG flows into the organ, and the change situation of its distribution can be quantitatively evaluated at the time of quantification by the evaluation index *h*, because of this non‐uniformity.

The strong correlation between vascular resistance during reperfusion and the evaluation index *h* suggests that it is possible to quantitatively predict the status of ischemic reperfusion injury before reperfusion, enabling reliable transplantation and ensuring the transplantation of DCD donors and marginally donated organs. This method is considered applicable not only to HOPE but also to NRP, NMP, and various surgeries involving ischemic time; further verification is necessary in the future. In the next preclinical study to evaluate the applicability of this technology, it is considered important to use experimental groups that include SCS, given the clinical use of HOPE; it is believed that introducing these methods will make differences in organ conditions more apparent.

In this study, there were no differences between the 2 experimental groups, WIT0 and WIT45. HOPE is a technique with the potential to restore organ function and offers hope for making organs transplantable after cardiac arrest. Previous studies on HOPE have reported a median fDWIT of 36 min, and it has been reported [45] that even grafts with a fDWIT as long as 50 min have resulted in successful transplants. In this study, experiments were conducted with a target WIT of 45 min. The fact that no correlation was observed between the results of the WIT45 group (including recovered grafts) and the healthy WIT0 group supports this conclusion. On the other hand, we believe that this method, which allows for the quantitative assessment of intra‐organ flow after reperfusion using indices during MP, will make a significant contribution to transplantation.

### 4.1 Limitations

It has been reported that the evaluation of the amount of FMN contained in the mitochondria that is lost to the perfusate reflects the state of liver mitochondria as an evaluation method for hypothermic perfusion conditions for organ preservation [34]. The proposed method evaluates ischemic injury from a flow perspective, focusing on the distribution of ICG fluorescence in blood vessels. However, it is also crucial to assess it in conjunction with FMN, which emphasizes metabolic characteristics. We believe that integrating FMN into the evaluation will be important in the future.

In addition, although evaluation by biopsy is also important, as shown in Figure 6, the condition of organs with the same preservation conditions varies locally, with differences between areas with perfusion and those without perfusion; therefore, histological comparison has not been conducted here. We believe that a comparative study with histological pathological evaluation is also important.

Another limitation of this experiment is that no transplantation was performed. Although the evaluation was conducted in a reperfusion model using diluted autologous blood to verify ischemia–reperfusion injury, and the usefulness of organ viability assessment methods in reperfusion models has been demonstrated [26, 27], it is necessary to conduct actual transplantation experiments to evaluate not only short‐term ischemia–reperfusion injury prediction but also long‐term prognosis. In addition, since the results were obtained in a large animal study using pigs, it is essential to verify the findings with clinical research data in the future.

In conclusion, the evaluation index *h* obtained by GLCM of ICG fluorescence images in organs during MP can predict portal vein resistance induced with ischemic reperfusion injury in organs before transplantation and is a method that can expand the possibilities of HOPE perfusion. In the future, we believe that by integrating indicators based on the flow patterns identified by this method with FMN data related to metabolism, we will be able to treat additional organs.

## Author Contributions

Y.O., H.O., S.S., and N.M. concept/design of the study; H.K., T.N., S.I., and N.M. performed the surgeries and experiments; Y.O., T.M., S.M., M.T., and M.F. performed machine perfusion experiments and data collection and analysis; Y.O., H.O., and N.M. drafting of the article; All authors have reviewed the results and approved the final version of the manuscript.

## Conflicts of Interest

The authors declare no conflicts of interest.

## Data Availability

The data that support the findings of this study are available on request from the corresponding author. The data are not publicly available due to privacy or ethical restrictions.
